# Quality and technical efficiency do not evolve hand in hand in Spanish hospitals: Observational study with administrative data

**DOI:** 10.1371/journal.pone.0201466

**Published:** 2018-08-02

**Authors:** Sophie Gorgemans, Micaela Comendeiro-Maaløe, Manuel Ridao-López, Enrique Bernal-Delgado

**Affiliations:** 1 Department of Management, School of Engineering and Architecture, University of Zaragoza, Zaragoza, Spain; 2 Institute for Health Sciences in Aragon (IACS), Zaragoza, Spain; 3 REDISSEC–Research Network for Health Services on Chronic Patients, Galdakao, Spain; University of Malta Faculty of Health Sciences, MALTA

## Abstract

**Objective:**

Recent evidence on the Spanish National Health System (SNHS) reveals a considerable margin for hospital efficiency and quality improvement. However, those studies do not consider both dimensions together. This study aims at jointly studying both technical efficiency (TE) and quality, classifying the public SNHS hospitals according to their joint performance.

**Methods:**

Stochastic frontier analysis is used to estimate TE and multilevel logistic regressions to build a low-quality composite measure (LQ), which considers in-hospital mortality and safety events. All hospitalizations discharged in Spain in 2003 and 2013, in 179 acute-care general hospitals, were studied. Four scenarios of resulting performance were built setting yearly medians as thresholds for the overall sample, and according to hospital-complexity strata.

**Results:**

Overall, since 2003, median TE improved and LQ reduced -from TE_2003_:0.89 to TE_2013_:0.93 and, from LQ_2003_:42.6 to LQ_2013_:27.7 per 1,000 treated patients. The time estimated coefficient showed technical progress over the period. TE across hospitals showed scarce variability (CV_2003_:0.08 vs. CV_2013_:0.07), not so the rates of LQ (CV_2003_:0.64 vs. CV_2013_:0.76). No correlation was found between TE values and LQ rates. When jointly considering technical efficiency and quality, hospitals dealing with the highest clinical complexity showed the highest chance to be placed in optimal scenarios, also showing lesser variability between hospitals.

**Conclusions:**

Efficiency and quality have improved in Spanish public hospitals. Not all hospitals experiencing improvements in efficiency equally improved their quality. The joint analysis of both dimensions allowed identifying those optimal hospitals according to this trade-off.

## Introduction

Health systems performance assessment (HSPA) has become a major priority in Europe as a way to strengthen health systems effectiveness [1]. In Spain, HSPA is getting momentum as a reaction to the deep financial crisis endured by the Spanish National Health System (SNHS) since 2010 [2]. In the context of the statutory SNHS where public hospital activity is purchased according to quasi-retrospective global bundled payments with no particular incentives for value improvement, and where the transfer of risks is not present (penalties for bad performance or bonuses for good performance are negligible), competition throughout HSPA benchmarking may serve as an alternative.

The most prevalent HSPA conceptual framework [3] suggests monitoring a set of dimensions such as *equity in access*, *sufficiency* in terms of financial endowment and *quality* as central focus, considering this last one as a nested matrix of the sub-dimensions *patient-centred care*, *efficiency* and *patient safety*. (See here for a graphical representation of the mentioned HSPA framework http://www.oecd.org/els/health-systems/health-care-quality-indicators.htm). Usually, HSPA dimensions are measured as if they were independent phenomena when, on the contrary, multiple trade-offs are possible; for example, quality and efficiency are usually reported separately when both, are closely related when aiming the maximization of healthcare value (i.e., increasing efficiency while improving quality) [4]. Actually, one of the major concerns when measuring and reporting technical efficiency (TE) lays on the frequent coexistence of hospitals equally efficient although exhibiting differences in quality [5–7]. More specifically, the direction of the association between quality of care and technical efficiency has shown mixed evidence. Some authors found that lower TE was associated with poorer quality outcomes [8] while others found that technically efficient hospitals were performing well with regard to quality [9], remaining unclear whether hospitals that successfully improve quality necessarily sacrifice production efficiency [10–11].

Recent evidence on the SNHS public hospitals reveals a considerable margin for hospital efficiency and quality improvement [12–13]. However, these studies do not consider both dimensions together. This study aims at jointly studying both TE and quality, and classifying public SNHS hospitals according to their joint performance.

## Methods and design

### Design and population

Observational cross-sectional study on virtually all hospital admissions discharged in two years, 2003 and 2013, in 179 acute-care public hospitals of the SNHS. The sample accounted for the 64% of the SNHS general hospitals and 83.3% of the overall admissions. Those non-existing hospitals in 2003 and hospitals with less than 30 episodes a year for those clinical conditions and procedures composing the measure of quality were excluded to avoid statistical noise.

### Main endpoints: Technical efficiency and quality

TE, measured as the distance from each observed hospital to a theoretical optimum, was estimated through Stochastic Frontier Analysis (SFA) using 3 inputs, 2 outputs and 2 contextual variables. Because of the funding, purchasing and reimbursement features of the strongly regulated SNHS [14], where prices are not playing the same role as in market-oriented health systems, our approach builds on physical measures (i.e., inputs). Input variables regarded i) *functioning beds* as proxy to physical capital and, ii) *full-time equivalent physicians* and iii) *full-time equivalent nursing staff*’ as human capital. In turn, two output variables were included: i) *discharges weighted by Diagnosis-Related Group* as a measure of risk-adjusted inpatient activity, and ii) *outpatient activity* (the sum of outpatient visits and emergency contacts). Time (year of observation) meant to capture the hospitals’ technological development (i.e., any eventual frontier displacement), and the teaching status of the hospital, meant to seize any eventual effect of academic environments, were included in the model as contextual factors.

Low-Quality (LQ) was defined as a weighted composite of events including: a) in-hospital mortality during the episode of admission of a cardiovascular event (Acute Myocardial Infarction, Angina or Cardiac insufficiency) or a scheduled intervention like Percutaneous Coronary Intervention or elective Coronary Artery Bypass Grafting; and, b) the presence of a safety event along the episode of admission, specifically, the presence of post-surgery pulmonary thromboembolism or a deep venous thrombosis (PTE), c) post-operative sepsis (POS), or d) the presence of bacteraemia associated to catheter (CRI). The composite aimed at seizing the overall hospital quality as included medical, surgical and nursing care. The indicators composing the LQ measure had been previously validated and reported [15–17].

### Analysis

TE was estimated through SFA a parametric method based on a panel regression model where the residuals are expected to represent stochastic noise and inefficiency. For that purpose an input orientation approach was used when estimating the Cobb-Douglas (CB) function of production. As compared to other functions, CB better controls multicollinearity, is easily transferrable to a linear regression model, and allows straightforward interpretation of the estimated parameters without major algebraic transformations [18–20]. Goodness of fit of CB was estimated using a Log-likelihood test [19]. Inefficiency would then result from the below distance of each observed hospital to the theoretical optimum estimate. In order to assess the evolution of TE (i.e., technical progress), the rate of progress was calculated by multiplying the model’s time coefficient by the elasticity of scale.

In-hospital risk-adjusted mortality and incidence of adverse events were calculated as the ratio of the observed to the expected cases, multiplied by the crude rate. The expected cases for each quality indicator were assessed by specifying multilevel logistic regressions, allowing for a general hospital cluster effect [21]. The estimation model included as covariates age, sex and the presence (or not) of the Elixhauser comorbidity conditions to reduce confounding phenomena across hospitals [22–23]. A single composite measure was finally obtained by weighting each quality indicator according to its death toll–indicator’s fatality rate to the overall death rate. TE and LQ, and their variation across hospitals (as coefficient of variation) were estimated for the overall sample of 179 hospitals.

Although the analyses used contextual variables and risk adjustment measures to reduce bias, subgroup analyses were additionally carried out to address the eventual effect on estimates of unobserved (latent) or not considered (e.g., not available) variables. For that purpose, SNHS acute-care hospitals were stratified into quartiles according to their actual treated complexity, measured throughout the overall sum of APR-DRGs weights per hospital. So, hospitals in this sample were clustered into four groups, as follows: group 1, included those hospitals with overall APR-DRG weight less than 4,860.3 (11 hospitals); group 2 with hospitals whose overall weight ranged from 4,860.3 to 10,253.8 (43 hospitals); group 3 included hospitals with overall weight ranging from 10,253.9 to 21,553.5 (56 hospitals); and, group 4 included those hospitals with overall complexity weight above 21,553.5 (69 hospitals).

**Fig 1:**
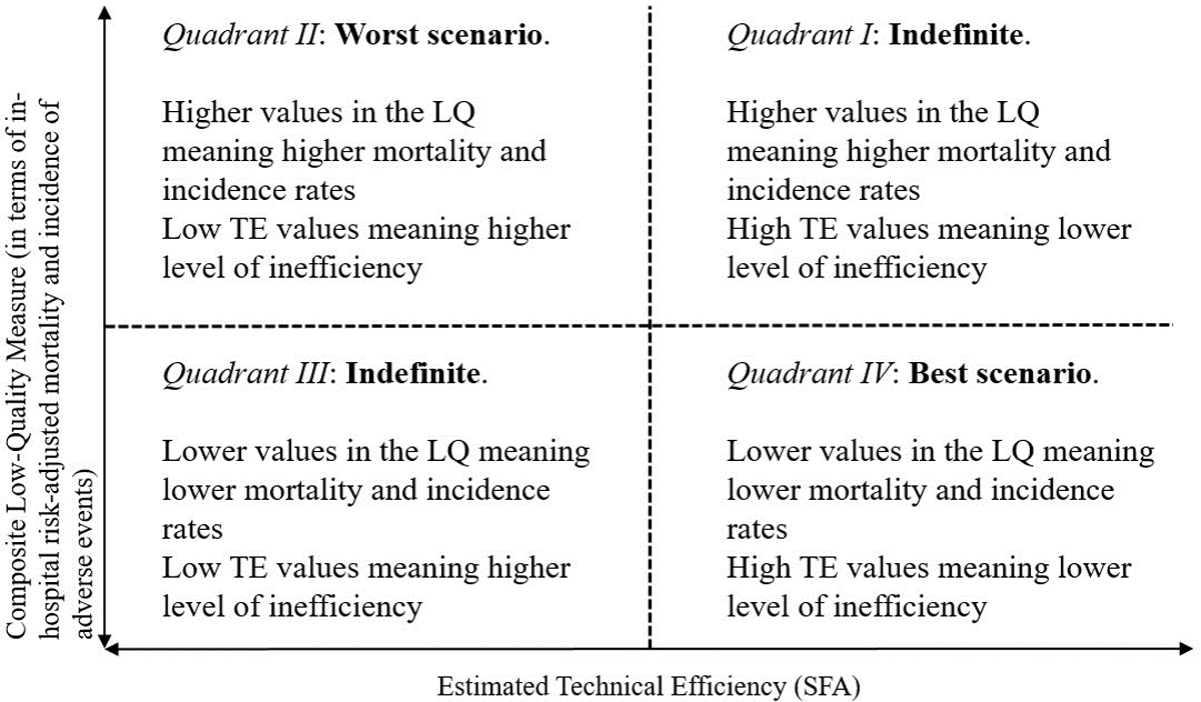
Definition of the classification quadrants.

Finally, hospitals were assigned to four scenarios, based on the joint performance of technical efficiency and quality. Quadrants were built upon the median values of TE (x axis) and LQ (y axis) for both, 2003 and 2013 (Fig 1). TE outcomes closer to 1 would represent more efficiency, and lower in-hospital LQ rates would represent better quality.

All specified models were coded and assessed using Stata v.14 and Frontier 4.1. Mathematical specifications are included in S1 Methodological Appendix.

### Sources of information

Three main data sources were used: 1) the Annual Hospital Survey (in Spanish, *Estadística de Establecimientos Sanitarios en Régimen de Internado*) from which inputs and outputs from the SFA were retrieved (https://www.msssi.gob.es/estadisticas/microdatos.do); 2) APR-DRGs weights using the APR-DRGs grouper licensed to the AtlasVPM group by 3M; and, 3) The AtlasVPM project data infrastructure, that contains administrative and clinical data from virtually all the hospitalizations produced in the SNHS since 2002. This data source was used in the extraction of quality indicators using the codes developed by the Atlas VPM project [24].

This study, observational in design, uses retrospective anonymized non-identifiable and non-traceable data, and was conducted in accordance with the amended Helsinki Declaration, the International Guidelines for Ethical Review of Epidemiological Studies, and Spanish laws on data protection and patients’ rights. The study implies the use of pseudonymised data, using double dissociation (i.e., in the original data source and once the data are stored in the database for analysis) and analyses and reporting are based on aggregated information, which impedes patients’ re-identification. The study was reviewed and approved by the Research Ethics Committee of Aragon (CEICA), who waived the need for written informed consent from the participants.

## Results

In 2013 (Table 1), the hospital activity (inpatient and outpatient care) accounted for 68 million episodes, a 17.7% more than in 2003. The increase was mainly due to outpatient contacts—18% (outpatient visits and emergencies) in contrast to the 9.9% increase of hospitalisations. In terms of inputs, overall, the amount of functioning beds decreased a 6.5% over the period, although more intensely in more complex hospitals (group 4), whose decrease reached a 9.1%. Fulltime physicians increased up to a 29.3% along the period, showing the highest increase (40.9%) in hospitals included in the third group (median to high complexity). Nursing staff increased overall, up to a 13.8%; by groups, in groups 2 and 3 nurses increased a 23%, while group 1 increased a 5%, and group 4 a 12%. Teaching status of hospitals did not vary along the period, with an 83.8% of teaching hospitals in both years. Descriptive figures for inputs and outputs showed low variability across hospitals within each group (Table 1).

**Table 1:** Inputs, outputs and quality indicators. Descriptive statistics.

INPUTS	Aggregate		Group 1		Group 2		Group 3		Group 4	
	2003	2013	2003	2013	2003	2013	2003	2013	2003	2013
**BEDS**	83932	78446	1177	1135	7184	7099	19220	18984	56351	51228
median	353	350	98	102	163	162	344	320	780	730
CV	0.76	0.71	0.20	0.14	0.32	0.31	0.33	0.35	0.39	0.36
**PHYSICIANS**	65418	84607	836	1048	4725	6422	13726	19345	46132	57792
median	256	367	73	96	117	145	225	300	599	794
CV	0.86	0.77	0.19	0.13	0.30	0.29	0.33	0.39	0.46	0.36
**NURSES**	173863	200076	2382	2502	13407	16501	36635	45171	121440	135903
median	633	776	202	217	305	374	611	710	1585	1837
CV	0.85	0.79	0.27	0.31	0.32	0.29	0.34	0.43	0.45	0.40
**OUTPUTS**										
**DISCHARGES**	2691538	2958233	34075	33996	235767	257861	618077	710324	1803619	1956053
median	11956	13306	2955	3089	5344	5770	10804	11694	23848	25769
CV	0.74	0.72	0.20	0.22	0.26	0.32	0.30	0.34	0.38	0.36
**OUTPATIENT VISITS**	55167310	65121777	1121363	1200740	6702785	7896399	14262629	17339700	33080533	38684938
median	256758	333049	100010	105469	154851	181547	246320	305593	420912	513118
CV	0.62	0.57	0.21	0.30	0.26	0.24	0.30	0.34	0.40	0.31
**QUALITY FIGURES**										
**Mortality in Cardiovascular events**										
Patient at risk (overall)	122004	130096	1109	1305	5875	6880	23807	28459	91213	93452
(median per hospital)	418	492	80	109	129	132	384	431	1315	1299
CV	0.97	0.85	0.72	0.44	0.48	0.62	0.47	0.54	0.47	0.36
Crude Rate (median)	66.30	40.44	109.76	39.22	93.75	55.56	70.72	39.05	52.79	36.59
CV	0.50	0.55	0.36	0.53	0.46	0.58	0.45	0.45	0.35	0.32
Risk-adj. Rate (median)	66.81	39.93	123.43	45.56	105.03	60.08	70.54	38.38	52.25	35.07
CV	0.64	0.81	0.54	0.74	0.58	0.81	0.55	0.54	0.39	0.37
**Central Venous Catheter Related Blood Stream Infection Rate (CRI)**										
Patient at risk (overall)	1932068	1681050	32405	26495	195899	171585	500969	441006	1202795	1041964
(median)	9569	8360	2922	2378	4456	3976	8497	7408	16370	14091
CV	0.64	0.60	0.17	0.18	0.29	0.28	0.30	0.25	0.34	0.29
Crude Rate (median)	0.95	1.28	0.41	0.45	0.67	1.02	0.80	1.03	1.50	1.65
CV	2.06	1.95	2.92	2.92	1.08	1.96	1.05	0.92	0.71	1.25
Risk-adj. Rate (median)	0.96	1.32	0.29	0.29	0.65	1.02	0.81	1.03	1.52	1.69
CV	2.09	1.99	2.95	2.95	1.16	1.98	1.08	0.96	0.72	1.25
**Postoperative Pulmonary Embolism or Deep Vein Thrombosis Rate (PTE)**										
Patient at risk (overall)	973385	1088014	14101	14449	86544	94492	230924	256868	641816	722205
(median)	4398	4842	1172	1263	2080	2181	3861	4509	8764	9546
CV	0.72	0.69	0.28	0.31	0.26	0.23	0.32	0.26	0.37	0.30
Crude Rate (median)	5.69	7.37	5.84	4.96	4.67	5.76	4.88	7.27	6.15	8.41
CV	0.45	0.40	0.54	0.64	0.62	0.51	0.46	0.37	0.31	0.30
Risk-adj. Rate (median)	5.66	7.37	6.10	5.00	4.30	5.41	4.84	7.24	6.28	8.53
CV	0.53	0.45	0.63	0.84	0.78	0.61	0.53	0.41	0.33	0.32
**Postoperative Sepsis Rate (POS)**										
Patient at risk (overall)	212517	173014	3326	2695	18868	15092	45509	37822	144814	117405
(median)	844	694	276	233	440	356	770	641	1888	1543
CV	0.81	0.76	0.33	0.34	0.34	0.35	0.43	0.34	0.44	0.38
Crude Rate (median)	0.93	3.75	0.00	0.00	0.00	2.11	0.87	4.42	1.81	4.35
CV	1.44	0.81	3.32	1.72	2.08	1.25	1.81	0.71	0.82	0.54
Risk-adj. Rate (median)	0.82	3.86	0.00	0.00	0.00	1.30	0.68	4.73	2.05	4.41
CV	2.17	1.21	3.32	1.74	2.88	1.55	2.47	1.14	0.95	0.68

Group 1 of hospitals: complexity case-mix out of APR-DRG less than 4860.27 (11 hospitals)

Group 2 of hospitals: complexity case-mix out of APR-DRG from 4860.28 to 10253.82 (43 hospitals)

Group 3 of hospitals: complexity case-mix out of APR-DRG: from 10253.83 to 21553.54 (56 hospitals) and, group of hospitals 4: complexity case-mix out of APR-DRG above 21553.55 (69 hospitals)

Crude and Risk-adjusted Rates are per thousand patients at risk

CV: coefficient of variation

In turn, quality figures also improved over the period of study; thus, for the in-hospital mortality due to cardiovascular events a 26.9 per thousand points reduction was observed between 2003 and 2013. By groups, lesser complex hospitals experienced the deepest decrease in mortality rate (77.9 per thousand points). Finally, for safety events, incidence remained almost the same for CRI, although slightly increased in the case of PTE (1.2 per thousand points) and POS (3 per thousand points), mainly in hospitals of median-higher complexity (group 3).

Looking separately at the performance dimensions (Table 2), from 2003 to 2013, the overall median TE improved, from 0.89 to 0.93 (frontier equals 1). Hospitals with middle to higher complexity (groups 2 to 4) improved their TE in four-hundredths, while hospitals in group 1, got closest to the optimum TE in 2013 (0.96). Very small variations in TE were observed, both across hospitals and over time, the lowest in group 1 (CV_2003_ 0.03 *vs*. CV_2013_ 0.04).

**Table 2:** Outcomes description.

TECHNICAL EFFICIENCY	Aggregate		Group 1		Group 2		Group 3		Group 4	
	2003	2013	2003	2013	2003	2013	2003	2013	2003	2013
median	0.89	0.93	0.91	0.91	0.88	0.92	0.90	0.94	0.91	0.95
CV	0.08	0.07	0.06	0.07	0.08	0.08	0.07	0.05	0.05	0.03
**COMPOSITE QUALITY MEASURE**										
median	42.55	27.68	62.92	30.83	54.02	30.58	34.99	23.90	32.78	25.47
CV	0.64	0.76	0.66	0.84	0.62	0.80	0.40	0.38	0.27	0.28

**Fig 2:**
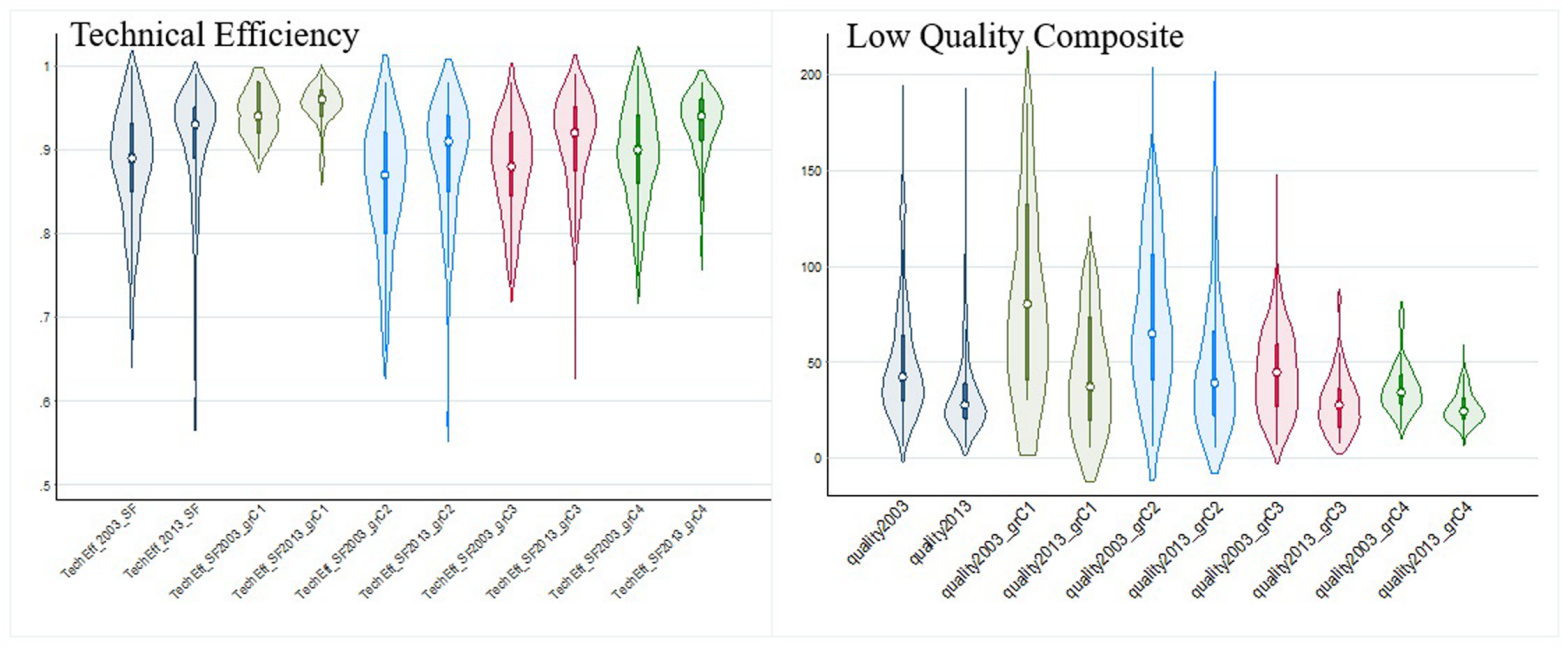
TE and LQ (overall and by hospital subgroups; 2003 and 2013). Violin graphs allow visualizing the distribution of quality (left) and efficiency (right) using a Kernel density function (the shape of the violin), and a boxplot representing the median value (hollow dots), the interquartile intervals (box) and percentiles 95^th^ and 5^th^ (the spikes).Group of hospitals 1: complexity case-mix out of APR-DRG less than 4,860.27 (11 hospitals). Group of hospitals 2: complexity case-mix out of APR-DRG from 4,860.27 to 10,253.82 (43 hospitals). Group of hospitals 3: complexity case-mix out of APR-DRG: from 10,253.82 to 21,553.54 (56 hospitals) and, group of hospitals 4: complexity case-mix out of APR-DRG above 21,553.54 (69 hospitals). Crude and Risk-adjusted Rates are per thousand patients at risk. CV: coefficient of variation.

In general terms, LQ reduced along the period with a remarkable decrease of negative events from 42.6 to 27.7 per 1,000 treated patients (median value). Unlike TE, the variation in LQ across hospitals was remarkably high overall, mainly in hospitals with low or median-low complexity, and was observed to increase along the years—from CV_2003_ 0.64 to CV_2013_ 0.76 (Fig 2 shows the magnitude of variation across hospitals -turnip plots, overall and by hospitals complexity).

Interestingly, the coefficient of the contextual variable of time showed technical progress over the period, so the rate of progress implied that an efficient hospital, with the same inputs, in 2013 would obtain 14.23% more outputs than in 2003.

No correlation was observed between TE and LQ, neither in 2003 nor in 2013, suggesting that not all hospitals experiencing improvements in efficiency equally improved their quality, nor *vice versa*. This finding was irrespective of the teaching status of the hospital.

**Fig 3:**
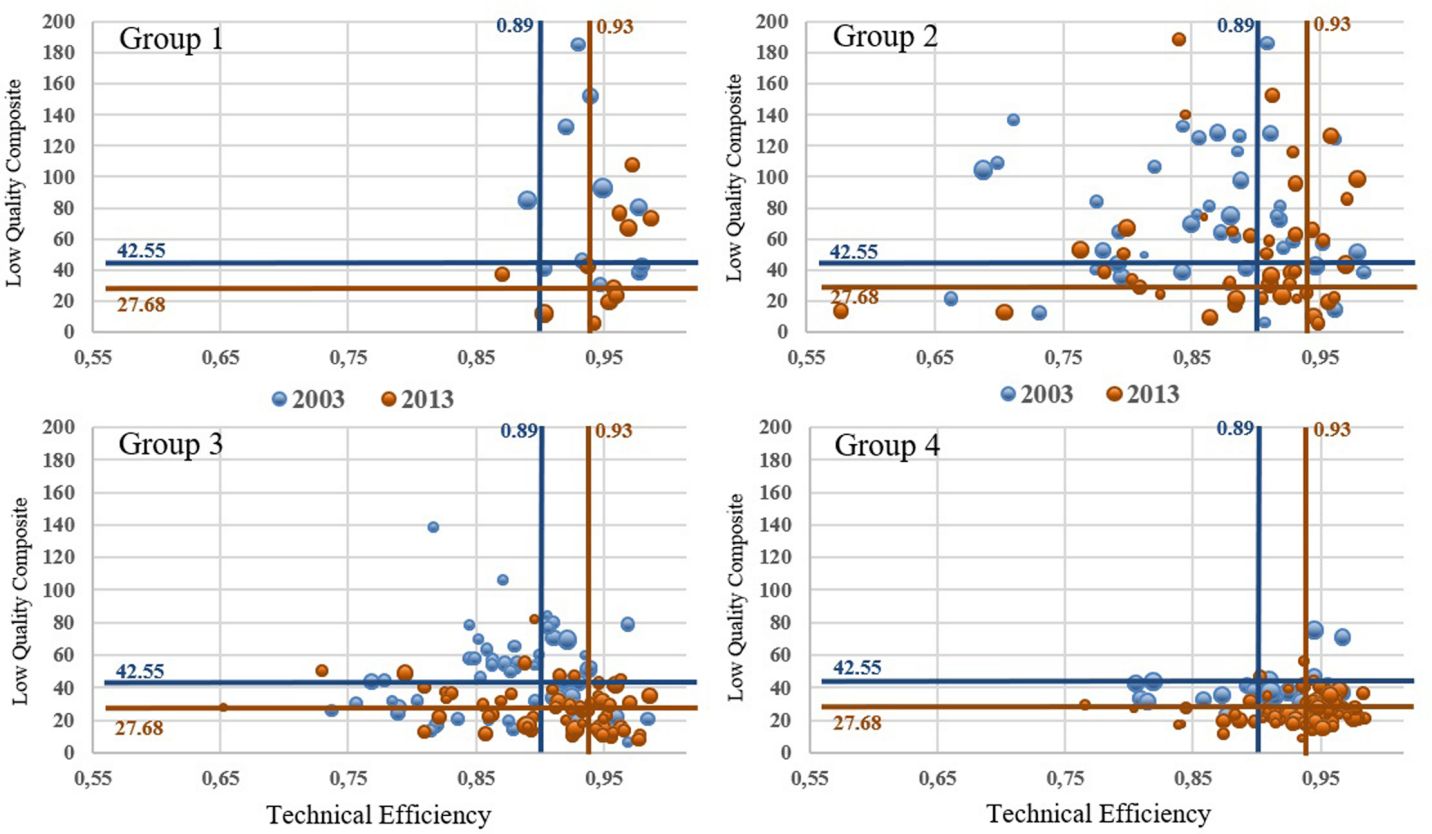
Dynamic joint performance assessment. Each bubble represents a hospital's outcome, blue bubbles account for 2003 figures and orange bubbles for 2013. Bubble size is related to the amount of functioning beds at each hospital. Lines placed at median values of TE and LQ delimits hospital's relative position. Low-quality (LQ) is measured in terms of per thousand patient at risk.

According to the graphical joint analysis (Fig 3), 47 out the 179 hospitals in the sample remained (19 hospitals) or evolved (28 hospitals) to the optimal scenario (highly efficient and high-quality hospitals); out of those, the 80.9% were hospitals with mild-to-high or high complexity -14 and 24 hospitals, respectively. On the contrary, 46 out of the 179 hospitals remained (19 hospitals) or evolved (27 hospitals) to the worst scenario (highly inefficient and poor-quality hospitals). Out of those, a 43.5% were mild-to-low complexity hospitals, and 41.3% were mild-to-high complexity hospitals -20 and 19 hospitals, respectively.

## Discussion

Acute hospitals in the SNHS experienced the improvement of both, efficiency and quality, between 2003 and 2013, although the overall improvement in quality was observed uneven across hospitals. Not all hospitals experiencing improvements in efficiency equally improved its quality, nor *vice versa*. Contradicting the commonplace that big hospitals are naturally inefficient, particularly if they are publicly tenured, and provide lower-quality, the more complex hospitals in our sample were placed at the optimal quality-efficiency trade-off scenario in 2013; nonetheless, lower-complexity hospitals showed to be more efficient (closer to the frontier) and experienced the deepest improvement in quality over the period.

In the Spanish context, where healthcare decision-making processes are decentralised to the Autonomous Communities, the heterogeneity of the accountability systems among the Regional Health Systems cause difficulties at creating a comprehensive strategy to assess efficiency across the SNHS [25]. On the other hand, although insistently demanded, the inclusion of quality in the efficiency models is rarely found, partly due to the difficulty in the selection of an overarching hospital quality indicator, comparable across different type of hospitals. Therefore, the joint assessment of efficiency and quality has rarely been approached in Spain. Those few studies [26–29] focused on the assessment to a particular region, used alternatively Data Envelopment Analysis (DEA) and included a variety of quality indicators (e.g., nosocomial infection as a non-quality indicator, PSIs, Inpatient Quality Indicators (IQIs), readmissions or patient perceived quality), concluding, so does this paper, that better efficiency does not necessarily mean higher quality. Only one of the papers [29] estimated TE introducing composite measure of IQIs and PSIs establishing an overall increase of efficiency in Andalusian hospitals. Similar conclusion was obtained for hospitals dealing with moderate complexity [30].

Regarding international literature, only few studies using SFA examined the relationship between hospital efficiency and quality [31–33] and they also revealed no systematic association between TE and quality of care, nor impact on hospitals’ ranking.

### Methodological caveats

To this respect, two elements should be discussed: on the one hand, on the use of SFA instead of DEA; and, on the other hand, on the use of a composite measure to seize overall hospitals quality.

When it comes to the utilisation of SFA vs. DEA, SFA may be more helpful to understand the future behaviour of the entire population of hospitals while DEA centered on individual behaviors [34], is being increasingly used in the analysis of efficiency and although it is still underused in the field of hospitals efficiency [35] and, finally, DEA health-care applications supposed a serious distortion because of the nature of the product in health care (neither homogeneous, nor unidimensional) [36]. SFA is a parametric method based on the principle of econometrics and the theories of the micro-econometrics, which use panel data regression models to estimate a conventional Cobb-Douglas function of production. In contrast, DEA is a non-parametric deterministic method which estimates efficiency based on multiple productivities.

An important strength of SFA lies on its ability to diagnose latent heterogeneities among hospitals and, as TE in SFA is measured by using the residuals of the regression, which are supposed to be caused by stochastic noise and inefficiency [37–38], estimations are less sensible to error than DEAs, owning a greater discrimination capacity between those efficient and inefficient units, while allowing statistical testing of hypotheses concerning production structure and degree of inefficiency [39]. However, SFA adds greater difficulty at modeling since its production frontier requires all outputs (or inputs) to be meaningfully aggregated into a single measure.

When it comes to seizing hospital quality, the use of composite measures is rather unusual, although individual measures only provide a partial picture of the overall quality of a hospital [40–45]. In this paper, providing an empirical weighting for those events composing ‘quality’ allows covering a broad range of quality elements concerning hospital activity (medical services, surgical services and nursing care), while allowing the identification of high-quality hospitals. Indeed, the discriminatory accuracy of the LQ measure (AUC and AUCw weighted) [21,46], reached figures above the 80%. Nevertheless, quality assessment methods and techniques using administrative data might endure well-known limitations, in particular, miss-classification biases that should be considered and controlled [47]. In the particular case of the indicators used in this study, their previous validation for Spanish hospitals [16] as well as the stratification of hospitals in complexity subgroups fosters a safer use in the measure of LQ.

### Implications

In the context of the SNHS, a system where public hospitals purchasing mechanisms lack of incentives to improve technical efficiency while increasing quality, monitoring the joint evolution of the technical efficiency and low-quality, providing hospital benchmarks, might foster performance improvement policies.

So, public purchasers, after monitoring hospital-providers evolution across TE vs. LQ trade-off scenarios, might compare the ‘distance’ between those hospitals and the benchmarks (those that remain in the optimal quadrant overtime) and nuance the ‘purchasing’ decisions accordingly.

## Conclusions

Acute hospitals in the SNHS experienced an improvement in both, technical efficiency and quality, between 2003 and 2013. Not all hospitals experiencing improvements in efficiency equally improved their quality, nor *vice versa*. The joint analysis of both dimensions allowed identifying those optimal hospitals according to the optimal TE vs. LQ trade-off.

## Supporting information

S1 Methodological Appendix Hereby Filename: S1 APPENDIX revised 20180412 submitted. (DOCX)
