# Supplementary material for: Quality and technical efficiency do not evolve hand in hand in Spanish hospitals: Observational study with administrative data
Source: PLoS One. 2018 Aug 2;13(8):e0201466. doi: 10.1371/journal.pone.0201466 (PMC6072019; doi:10.1371/journal.pone.0201466)
Supplement: S1 Methodological Appendix — (DOCX) [file pone.0201466.s001.docx]

**METHODOLOGICAL APPENDIX**

***Stochastic frontier especification model***

The production technology is being considered by using the production possibilities set:


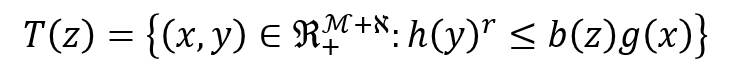
 (1)


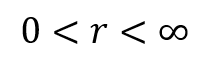
Where: *r* is an unknown parameter


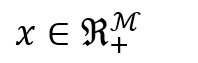
 is a vector of input quantities


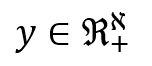
 is a vector of output quantities, and


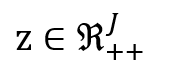
is a vector of exogenous variables measuring characteristics of the production context.

These properties ensure that the production possibilities set satisfies standard regularity properties (i.e. weak essentiality, free disposability of outputs and inputs, and closedness of output and input sets), they also mean that *r* can be interpreted as the elasticity of scale. Production technologies can also be represented using Shephard (1970) input distance function giving the largest factor by which the input vector can be scaled down while holding the output vector fixed. The logarithms of the input function distance is:


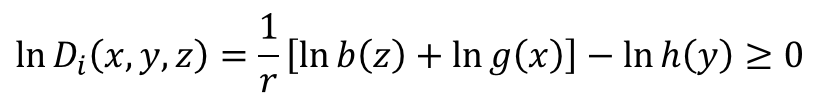
 (2)

In the first step of an econometric estimation of the production technology a Cobb-Douglas function can be used with unobserved errors representing statistical noise. The objective of this second step is to rewrite the input distance function in the form of a conventional stochastic frontier model, solving the endogeneity problem, as follows:


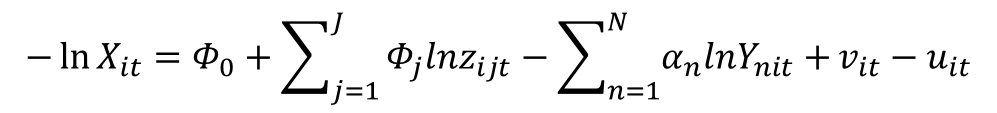
 (3)

Where: *Y_it_* and *X_it_* are the aggregate quantities defined by *Y_it_* ≅ *Y* (vectors of outputs quantities_it_) and *X_it_* ≅ *X* (vectors of inputs quantities_it_)

*z_it_* characterized the production context

*v_it_* is a composite error representing statistical noise~ iid N*(0,σ^2^_v_)* and

*u_it_* is a technical inefficiency effect ~ iid N^+^(0, $\sigma_{u}^{2}$). If u=0 then the hospital is 100% efficient and if u>0 the there is some inefficiency.

As no price data were available, linear programming (LP) methods were used to construct X_it_: X(x_it_) without requiring any explicit assumptions concerning functional forms or associated weights. In this paper, these aggregate inputs were used to create the dependent variable in (3). If there is no variation in the output vector (Y=1) a new approach (O’Donnell, 2010) considered then the LP is equivalent to the standard Data Envelopment Analysis (DEA) LP used to compute the reciprocal of the it-th technical efficiency score under the assumption of constant returns to scale.

Finally, Maximum Likelihood were used to estimate the Cobb-Douglas specification. These estimates are reported in table 1 and all of them have the expected signs and are significantly different from zero at the 1 % level (except Teaching status at the 5% level).

Table 1. Estimated model parameters.

| **-ln(x)** | **Coefficient** | **95% CI** | **p-value** |
| --- | --- | --- | --- |
| Time (t) | 0.1463 | (0.1037 0.1889) | 0.000 |
| Teaching status | -0.0712 | (-0.1337 -0.0088) | 0.026 |
| -ln (adjusted hosp. discharges) | 0.8959 | (0.8213 0.9706) | 0.000 |
| -ln (outpatient visits and emergencies) | 0.1264 | (0.0323 0.2205) | 0.009 |
| intercept | 8.4447 | (7.8528 9.0361) | 0.000 |

***Composite Quality Measure-* CQ**

In-hospital mortality in coronary processes and three PSI were used for the purposes of this study. To construct the CQ, to obtain an adjusted rate we transformed the crude rate multiplying it to the observed against expected approach, being the observed the number of cases (cardiovascular care and adverse events) at each hospital under study, and the expected, the predicted cases from a logistic multilevel regression considering as endogeneous variable the presence or not presence of the event and, as covariates, age, sex and the whole Elixhauser set of comorbidities of each patient.

$$Expected value=logit\left( P_{ij} \right)=ln\left( \frac{p_{ij}}{1-p_{ij}} \right)=\gamma_{00}+\sum_{k=1}^{K} \gamma_{k0}x_{kij}+u_{oj}+\varepsilon_{ij}$$

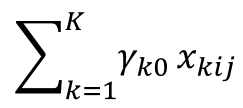
Where: are the covariates of each episode

*u_oj_* the aleatory effect of the hospital j ~ *iid N*(0, σ^2^*_u_*)

ε*_ij_*  the aleatory effect of the episode i in hospital j ~ *iid N*(0, σ^2^_ε_)

The in-hospital adjusted rates were afterwards weighted as follows: 60.6% for coronary care; 12.1% for PSI12; 21.2% for PSI13 and 6.1% for PSI07.
